# Surgical resection and reconstructive techniques using autologous femoral head bone-grafting in treating partial acetabular defects arising from primary pelvic malignant tumors

**DOI:** 10.1186/s12885-019-6196-x

**Published:** 2019-10-18

**Authors:** Wei Sun, Pengfei Zan, Xiaojun Ma, Yingqi Hua, Jiakang Shen, Zhengdong Cai

**Affiliations:** 1 Department of Orthopedic Surgery, Shanghai general hospital affiliated to Jiaotong University, No.100 Haining Road, Hongkou District, Shanghai, 200080 China; 2 Department of Orthopedic Surgery, Second affiliated hospital of Zhejiang University, Hangzhou, China

**Keywords:** Bone malignancy, Acetabulum, Reconstruction, Prosthesis survival

## Abstract

**Background:**

The aim of this study is to present and evaluate surgical resection and reconstructive techniques using autologous femoral head bone-grafting in treating partial acetabular defects arising from primary pelvic malignant tumors.

**Methods:**

From January 2009 until January 2015, a total of 20 primary pelvic malignancy cases involving the acetabulum were retrospectively investigated. Surgical resections and reconstructions were conducted based on the type of the tumor with custom osteotomy guides and autologous femoral head bone-grafting. In all cases, prosthesis survival period, complication occurrence, and clinical outcomes data were collected and analyzed.

**Results:**

Thirteen male and 7 female patients with an average age of 48 years old (range 23-69 years old) were followed for a median of 69 months (range 48-112 months). Of these cases, 17 included chondrosarcomas and 3 additional patients with a malignant giant cell tumor of bone (MBGCT) as proven by pathology. During follow-up, 3 cases of chondrosarcoma recurred (15%), of which two cases received hemi-pelvic amputation, 1 case of MBGCT relapsed and developed pulmonary metastases. Two cases of acetabular prosthesis with an impending dislocation received closed reduction followed by 6 weeks of hip abduction brace fixation. One case of prosthesis loosening was revised. In another case a deep infection occurred with debridement and prosthesis removal. Musculoskeletal Tumor Society 1993 (MSTS-93) score was utilized to conduct functional evaluation: 13 cases were good, 6 were average and one was poor.

**Conclusion:**

The precision of the osteotomies performed is likely crucial for this type of reconstructive strategy to be successful. The use of custom guides for acetabular osteotomies and femoral head reconstruction can improve functional outcomes with relatively low complications at the intermediate length of follow-up.

## Background

Treatment of malignant pelvic tumors involving the acetabulum requires a delicate balance between sufficient resection and functional reconstruction. The complicated anatomical structures and biomechanics of the pelvis as well as the difficulties in early diagnosis increase the surgical risks. Additionally, reconstructing the pelvis after extensive resection is challenging, as suboptimal functional recovery, multiple complications, and a higher rate of distant metastases after reconstruction seriously impede the patients’ quality of life. In cases with severe complications, these procedures impose a threat to life. Thus, the reconstruction of acetabular defects has proven to be a technically difficult surgical procedure [1–4].

Case reports indicated acceptable functional outcomes after resection of a pelvic tumor with no reconstruction thus leaving a flail hip [5, 6]. However, hemi-pelvic resection and allograft prosthetic composites have recently grown in popularity and area commonly used surgical techniques for the treatment of acetabulum involved pelvic malignant cases especially for younger patients [7–10]. The reconstruction can be clinically divided into biological and non-biological reconstruction. Non-biological reconstruction involves hemi-pelvic replacement with a metallic prosthesis. The biological reconstruction contains allogeneic hemi-pelvic replacement, proximal femoral eversion implantation and inactivation of the tumor bone for implantation, which is an optimal choice for primary malignancy with anticipated better prognosis considering the long-term functional recovery and biomechanical stability [11, 12].

A resource shortage for suitable allogeneic hemi-pelvic replacement, longer postoperative hospital stays, high infection rate, the probability of bone resorption and fracture, bone non-union and tumor recurrence have overwhelmingly hindered the techniques’ development. Recently, the eversion of the proximal femoral head for transplantation has prevailed among the biological reconstruction techniques [4, 13]. Of the non-biological reconstructions, saddle-shaped prosthesis has been rarely used due to a high complication rate resulting in lower average functional scores [14–16]. At present, the most common clinical approach is the metal prosthesis reconstruction. However, a higher perioperative complication rate and subjacent long-term survival rate have imposed difficulties on revision [16–19]. Thus, the optimal choice concerning prosthetic reconstruction after hemi-pelvic resection remains controversial.

Whether hemi-pelvic reconstruction is necessary in cases of pelvic tumor resection in which the partial acetabulum remains in place, is questionable. Biau DJ and colleagues [20] reported thirteen patients with a malignant pelvic lesion who underwent resection, followed by reconstruction using an ipsilateral femoral autograft and total hip replacement. In this reconstruction, the femoral head is connected to the lower ilium, the distal femur is connected to the pubis or ischium, and a pelvic reconstructive plate is utilized to rebuild the inner pelvic ring structure. Finally, total hip replacement or an acetabular reinforcement ring is applied to reconstruct the hip joint. In this way, reconstruction using a hemi-pelvic prosthesis is not preferred. This technique has proven to be associated with better healing of the bone structures, faster functional recovery, and lower costs, compared to hemi-pelvic prosthesis reconstruction.

In the present study, clinical information of 20 patients with a primary malignant pelvic tumor involving the acetabulum was retrospectively collected and reviewed. The three main purposes of the study are: a) to present the individually used reconstructive techniques after resection of the acetabular tumor; b) to discuss the indications of this type of surgery and reconstructive techniques; and c) to elucidate functional outcomes and survival of the reconstructions being used.

## Methods

A retrospective study was conducted after approval was obtained from the institutional review board of our hospital. All patients with the diagnosis of a primary pelvic malignant tumor involving the acetabulum undergoing surgical treatment in our institution were reviewed. The following inclusion criteria were considered: patients with a primary pelvic malignant tumor involving the acetabulum but without involvement of nerve and blood vessels, and of whom physical health could tolerate surgical intervention. Also, according to the principal of tumor resection, the possibility to preserve part of the acetabulum and presence of normal bone quality without obvious osteoporosis, therefore allowing primary stability after bone transplantation and construction. The following exclusion criteria were applied: metastatic bone tumor, patients with less than 2 years’ follow-up, and patient with a tumor involving the acetabulum having undergone previous curettage, resection, radiofrequency ablation or cryosurgery.

According to the inclusion and exclusion criteria, from January 2009 until January 2015, a total of 20 patients were included for analysis, of whom 13 were males and 7 were females, with an average age of 48 years old (range 23-69 years old).

The main manifestation of the malignancy is pain of the hip joint. Therefore, the visual analogue scale (VAS) was engaged to quantify the pain degree. In our patient database, 7 cases scored VAS 2; 11 cases scored 6 and two cases scored 8. Regarding other symptoms, 7 cases presented with mild clinical symptoms such as occasional pain after walking. Eleven cases had pain during walking with recovery in rest, where no symptoms were being observed when stretching without weight-bearing. Among these 11 cases, two were unable to walk properly after a long walk, where symptoms persisted and only slightly improved after resting. Five cases were presented with obvious nocturnal pain requiring oral analgesic drugs. Lastly, two cases suffered of pain in standing position while using braces as walking assistance.

All patients underwent preoperative imaging in the form of X-ray, Computed Tomography (CT) scan and Magnetic Resonance Imaging (MRI). These imaging techniques mainly revealed osteolytic destruction. In 14 cases (70%, 14/20), an obvious soft tissue mass was made visible without blood vessels and nerves involvement. All 20 cases involved bone destruction of the acetabulum, of which 5 cases (25%, 5/20) the articular surface, 17 cases (85%, 17/20) with obvious calcification in the destruction, and 11 cases (55%, 11/20) with obvious expansive growth.

Preoperative biopsy was conducted in each patient to confirm tumor pathology: chondrosarcoma was detected in 17 cases (85%, 17/20); of these 5 cases were grade 1(25%, 5/20) and 12 cases grade 2(60%, 12/20). MBGCT was detected in 3 cases (15%, 3/20).

After confirmation of the tumor diagnosis by imaging and pathology, all patients received preoperative angiography examination to detect the tumor’s blood supply, followed by vascular embolization. All tumors included in this study were chemotherapeutic insensitive types, thus no preoperative chemotherapy was given.

**Fig. 1:**
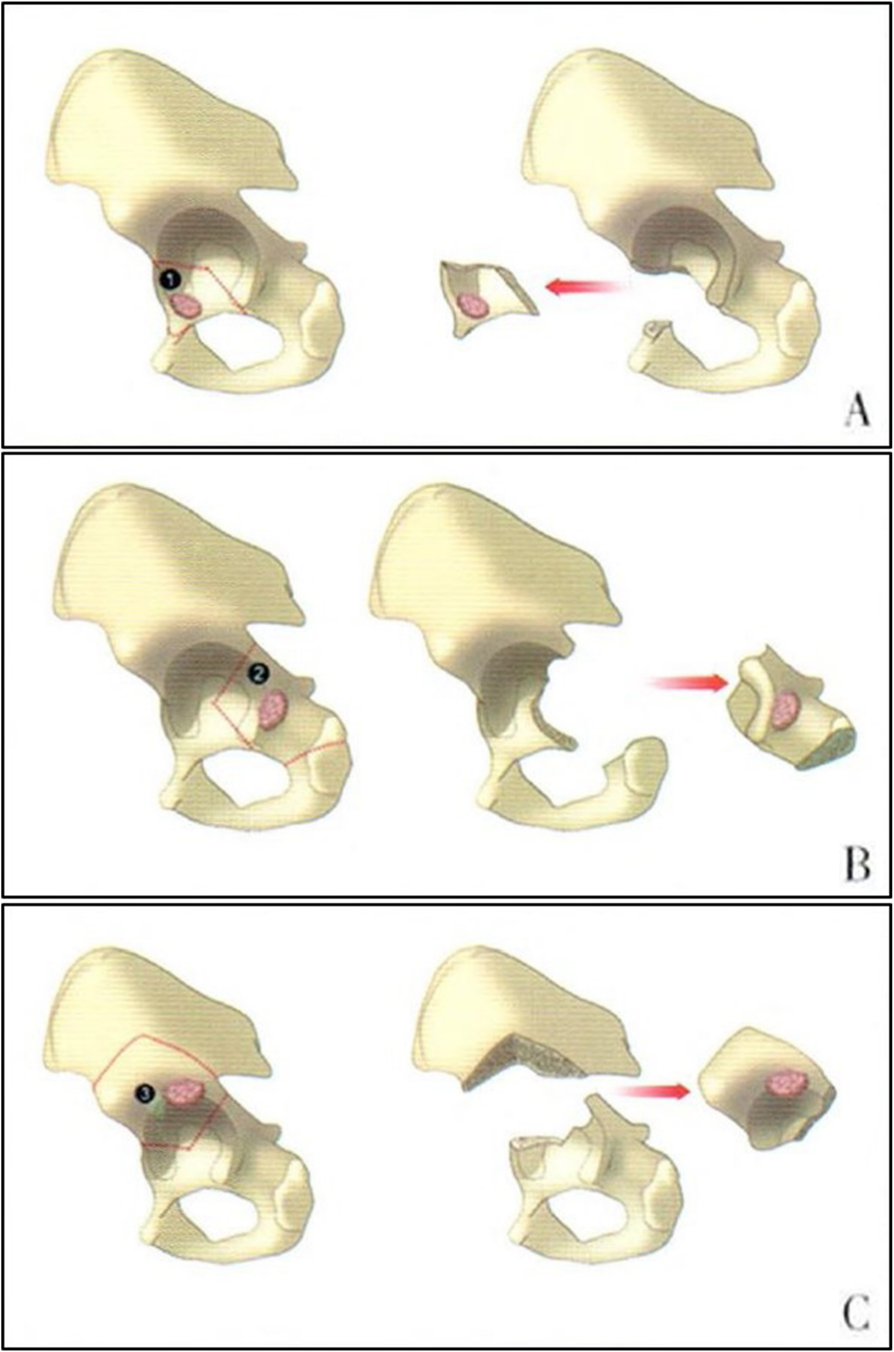
Diagram of acetabulum involved tumor classifications according to the anatomy and reconstruction techniques. Type **a**, pubic ramus tumor with anterior edge of acetabulum involved. Type **b**, Tumor with posterior column of the acetabulum involved. Type **c**, iliac tumor with superior edge of acetabulum involved. (Figure was drafted by our research group)

All surgeries were performed under general anesthesia, and patients were lying on their sides on the operation table. Different surgical approaches were used based on the tumor’s anatomy and reconstructive techniques after resection, as explained here and illustrated in Fig. 1.

Type A: A tumor of the pubic ramus with involvement of the anterior edge of the acetabulum. Combined ilium and groin Smith-Petersen (S-P) approach was utilized to perform the procedure. Attention was paid specifically to protect the femoral vessels and nerves during exposure of the ilium and groin area, and the spermatic cord in male patients.

Type B: A tumor located on the posterior column of the acetabulum. In this procedure, a Kocher-Langenbeck (K-L) approach was deployed with an incision of the medial femoral sciatic branch was occasionally needed, partially depending on the length of the tumor along the posterior column of the acetabulum. It is important to note the deep location of the tumor on the posterior column of the acetabulum, which could theoretically cause rupture of the tumor and contamination during traction. Thus, adequate incision and careful exposure was highly important.

Type C: A tumor of the ilium with involvement of the superior edge of the acetabulum. In this type of tumor, a Smith-Petersen approach was optimal to expose the tumor. Attention was being paid to protect the superior gluteal vessels and the sciatic nerve around the greater sciatic notch. A blunt metal barrier was occasionally placed to prevent injuries when performing osteotomy.

**Fig. 2:**
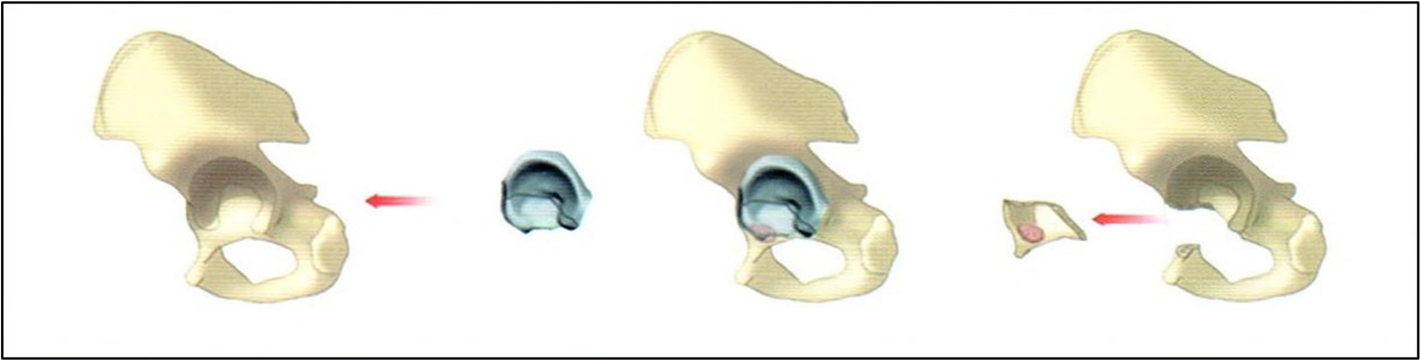
A schematic drawing of the osteotomy template for a Type A tumor. (Figure was drafted by our research group)

After tumor resection, individually designed reconstruction of the acetabular defects is of crucial importance. We made use of an individually designed 3D printing acetabular template for each patient based on the preoperative CT and MRI images, and marked the osteotomy lines on the template. The template was accurately placed according to the position of the fossae ovalis, after which osteotomy was performed based on the osteotomy template (a type A tumor osteotomy template was shown as Fig. 2).

Type A: 9 cases. After resection of the pubic ramus, a pelvic reconstructive plate was applied to rebuild the integrity of the pelvic ring. The autogenous femoral head transplantation was determined based on the position of the remaining acetabulum, and fixed with cancellous screws. Biological acetabular prosthesis was implanted after reconstruction of the anterior acetabulum.

Type B: 5 cases. After resection of the posterior column of the acetabulum, autogenous femoral head transplantation was conducted to bolster the defects in the posterior acetabulum and fixed with longer cancellous screws. Hereafter, biological acetabular prosthesis was implanted. With large acetabular defects, the initial stability of the femoral head implantation was poor. In those cases, an acetabular reinforcement ring could be applied before implanting the autogenous femoral head graft. A cemented mortar cup was inserted after filling the gap with cancellous bone particles.

Type C: 6 cases. After resection of the ilium, the defect of the acetabulum is located in the acetabular loading area. To ensure initial stability of the prosthesis, the rotated acetabular center needs to be moved slightly upwards. An acetabular reinforcement ring was inserted following autogenous femoral head implantation and cemented mortar cup insertion. It is important to implant the femoral side prosthesis with a higher offset, with the purpose of increasing the tension of the soft tissues and maintaining joint stability.

Drainage was removed in case of a drain volume less than 100 ml per day or in 72 h. No chemotherapy was given in this cohort. One week postoperatively, patients were mobilized to sit up beside the bed. After 4 weeks, patients were allowed to stand with double crutches to start practicing walking.

Patients received outpatient follow-up at 6 weeks, 3 months, 6 months, 1 year and following every year postoperatively. Pelvic CT and X-ray were performed to evaluate the location and stability of the prosthesis. Pulmonary CT was performed to detect possible pulmonary metastases. Systemic isotope bone scan was performed every 6 months after surgery to assess local control of the tumor.

Functional outcomes were measured using the 1993 Musculoskeletal Tumor Society Scale (MSTS-93) [21]. Pain, function, emotional acceptance in upper and lower extremities, upper limb factors (hand positioning, dexterity, and lifting ability) and lower limb factors (support, walking and gait) were scored adding up to a total of 30 points. A score of 80-100% was defined as excellent, 60-79% as good, 40-59% indicating average, and less than 40% was defined as poor.

## Results

Patient demographic data are shown in Table 1. All 20 patients included in the cohort underwent successful surgeries and none died in the post-operative period during hospitalization. The operation time was 3.0 to 4.5 h, with an average of 3.5 h. The perioperative bleeding was 800 ml to 3200 ml among the patients, with an average of 1300 ml. The follow-up time postoperatively was 48 to 112 months, with a median follow-up of 69 months.

**Table 1:** Patients’ demographic characteristics

NO.	Age (years)	Side	Diagnosis	Size of tumor(cm)	Type	Follow-up (months)	Local recurrence	Metastasis	Retention of original implant	Time to bone union	Complications	MSTS93 score
1	40-50	R	Chondrosarcoma	10*8*6	A	72	N	N	Y	6	None	19
2	20-30	L	Chondrosarcoma	7*4*5	B	60	N	N	Y	6	None	22
3	40-50	L	MBGCT	9*7*4	B	67	N	N	Y	6	None	21
4	50-60	L	Chondrosarcoma	12*6*8	A	56	Y	N	Y	12	Relapse, hemi-pelvic amputation	19
5	30-40	R	Chondrosarcoma	7*6*4	A	48	N	N	Y	6	None	23
6	50-60	R	Chondrosarcoma	10*8*8	A	50	N	N	N	None union	Prosthetic loosening	20
7	40-50	R	Chondrosarcoma	12*9*9	C	62	N	N	Y	12	Dislocation	17
8	60-70	L	Chondrosarcoma	8*8*7	B	70	N	N	Y	8	None	15
9	50-60	R	MBGCT	15*11*8	C	112	N	N	Y	8	None	19
10	50-60	L	Chondrosarcoma	8*6*6	A	69	N	N	Y	6	None	22
11	40-50	R	Chondrosarcoma	11*9*5	A	82	Y	N	N	9	Relapse	19
12	30-40	L	Chondrosarcoma	15*10*6	B	55	N	N	Y	6	None	23
13	40-50	R	Chondrosarcoma	13*8*6	C	57	N	N	Y	9	Dislocation	16
14	40-50	L	Chondrosarcoma	10*10*7	B	63	N	N	Y	6	None	16
15	50-60	R	MBGCT	13*12*8	A	49	Y	Y	N	9	Relapse and metastasis	16
16	50-60	L	Chondrosarcoma	11*5*6	A	83	N	N	Y	8	None	22
17	40-50	R	Chondrosarcoma	12*8*8	C	90	Y	N	N	9	Relapse, hemi-pelvic amputation	17
18	60-70	R	Chondrosarcoma	6*6*7	A	58	N	N	Y	6	None	23
19	50-60	L	Chondrosarcoma	16*11*9	C	93	N	N	N	None union	Infection	7
20	40-50	R	Chondrosarcoma	9*11*7	C	77	N	N	Y	6	None	20

*M* Male, *F* Female, *R* Right, *L* Left, *MBGCT* Malignant bone giant cell tumor; MSTS93: 1993 Musculoskeletal Tumor Society Scale

Among the 20 patients, three cases with grade 2 chondrosarcoma had a recurrence of the tumor at 7, 13, and 14 months respectively. One was treated with local tumor re-resection and the other two received hemi-pelvic amputation. One case of MBGCT recurred with pulmonary metastases at 9 months postoperatively, and received hemi-pelvic amputation and thoracoscopic metastases resection. This patient was followed for 49 months with no sign of relapse or metastasis.

Functional scoring was performed at 12 months follow-up using MSTS-93, which scored 7 to 23 in the cohort with an average of 18.8 points. Thirteen cases were evaluated as good, 6 cases were average, and 1 case was poor. The latter case experienced deep infection postoperatively accompanied by pain and walking disability. The prosthesis was removed in this particular case.

**Fig. 3:**
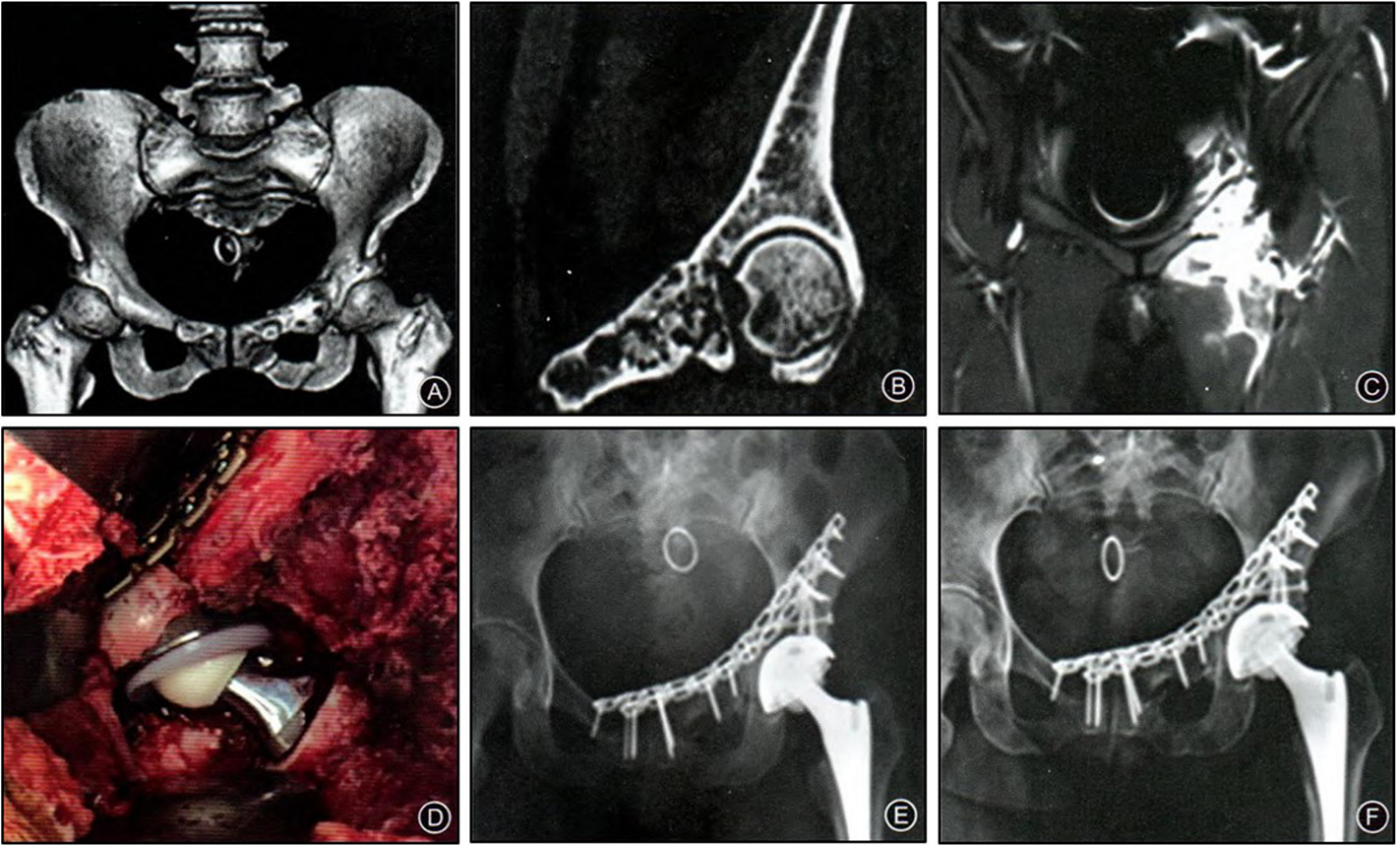
A 56y female patient diagnosed with Type A tumor (chondrosarcoma). **a**. Preoperative three-dimensional CT shows left pubic ramus tumor with anterior edge of acetabulum involved; **b**. Preoperative coronal CT shows pubic ramus tumor with anterior edge of acetabulum involved; **c**. Preoperative MRI T2 W1 shows enhanced signal on the pubic ramus and anterior edge of acetabulum; **d**. Intraoperative autogenous femoral head implantation and reconstruction of the acetabulum; **e**. Postoperative X-ray shows the reconstruction and prosthetic position; **f**: 1y postoperative pelvic X-ray shows the reconstruction and prosthetic position

**Fig. 4:**
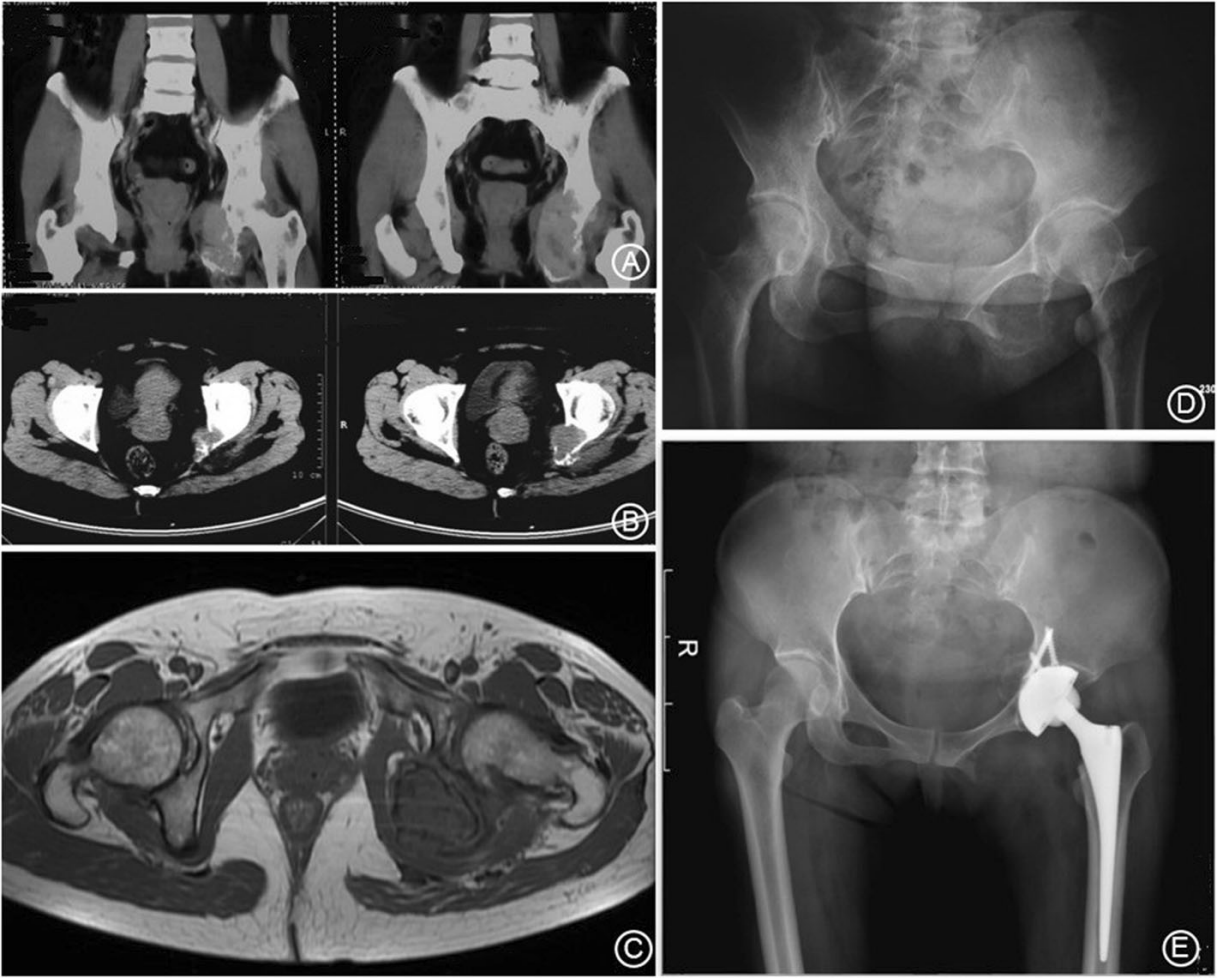
A 37y female patient diagnosed with Type B tumor (chondrosarcoma). **a**. Preoperative coronal CT shows a tumor with left posterior column of the acetabulum involved; **b**. Preoperative cross-sectional CT shows the tumor with posterior edge of the acetabulum involved; **c**. Preoperative MRI shows low signal on the posterior edge of acetabulum; **d**. Preoperative pelvic X-ray examination shows a tumor with left posterior column of the acetabulum involved; **e**. 1y postoperative pelvic X-ray shows the reconstruction and prosthetic position

**Fig. 5:**
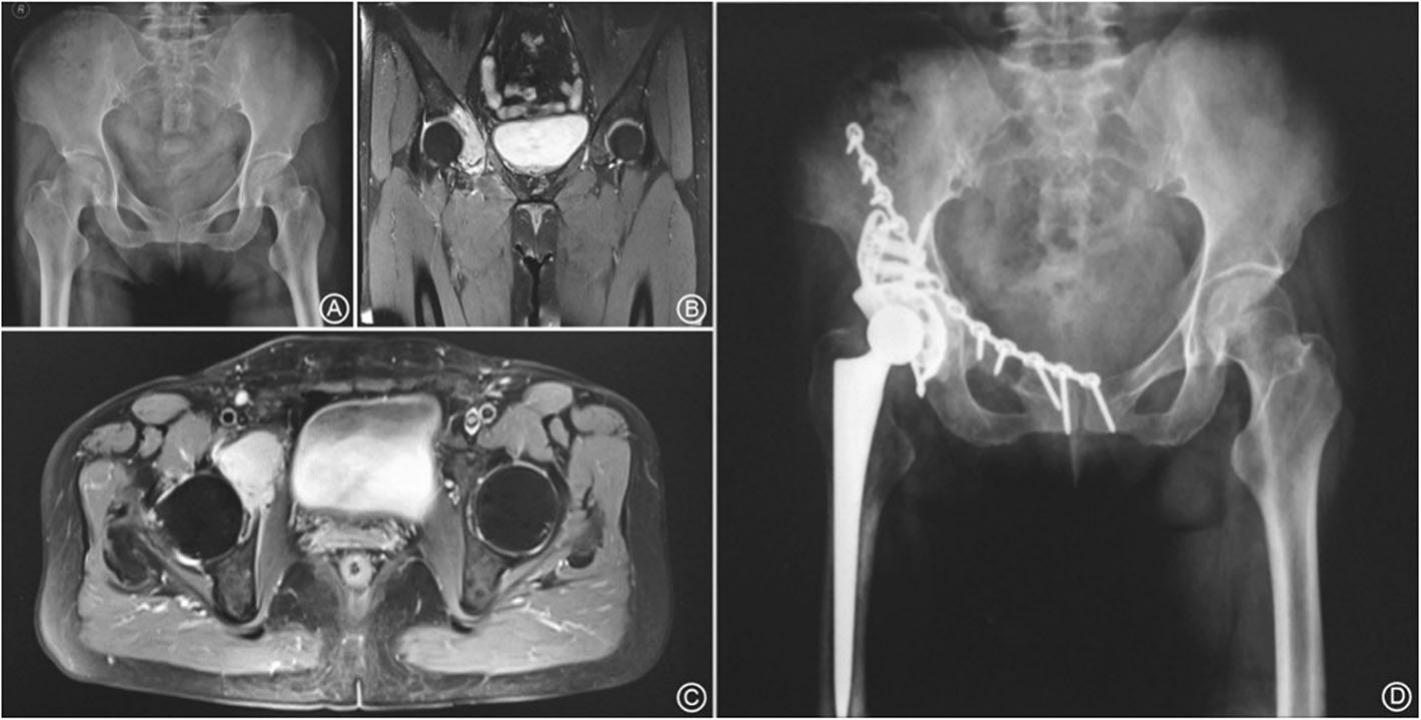
A 57y female patient diagnosed with Type C tumor [malignant giant cell tumor of bone (MBGCT)]. **a**. Preoperative pelvic X-ray shows a right ilium tumor with the involvement of the superior edge of the acetabulum; **b**. Preoperative coronal MRI shows enhanced signal on the superior edge of acetabulum; **c**. Preoperative cross-sectional MRI shows enhanced signal on the superior edge of acetabulum; **d**. 1y postoperative pelvic X-ray shows the reconstruction and prosthetic position

Of the 9 Type A cases in total, 8 were evaluated as good and one case was average. Resection of the tumor had little influence on the loading area of the acetabulum, which resulted in initial stability of the reconstruction using bone grafts (A standard case is presented as Fig. 3). Of the five Type B cases, three cases were evaluated as good and two as average. These patients underwent a resection of posterior column of the acetabulum. The initial stability was poor after bone graft reconstruction, resulting in a delayed onset of functional exercise (A case is shown as Fig. 4). Of the 6 Type C cases, two were evaluated as good; three cases were average and one scored poor. In these patients, reconstruction of the loading area of the acetabulum was necessary and soft tissue defects were large, both factors leading to an instable joint, resulting in poor functionality (A case is shown as Fig. 5).When comparing the functional scores between the tumor types, the scores in Type A patients were significantly better than those in Type C group (*p* = 0.032); no significant differences were detected in the other pairwise comparisons, B vs. C and A vs. B (*p* > 0.05). Postoperative imaging indicates that 18 patients in the cohort had successful bone graft union. One case developed a deep infection and in another case, loosening of the acetabular prosthesis occurred, resulting in non-union of the bone graft.

Five months after surgery, one patient with chondrosarcoma experienced walking pain without resting pain, but the complaint aggravated progressively. In this case, loosening of the acetabular prosthesis was detected on X-ray examination. The patient received a hemi-pelvic prosthesis revision. Two cases experienced hip joint dislocation 7 days and 12 days postoperatively respectively. These patients were treated with a closed reduction under general anesthesia, followed by an abduction brace for fixation during 6 weeks. Neither experienced further dislocation. One patient with chondrosarcoma developed fever, further examination by incision showed sinus formation with purulent secretion and bacterial culture was positive for *Staphylococcus aureus*. This patient received debridement and removal of the prosthesis.

## Discussion

In this study, a series of 20 patients with chondrosarcoma or MBGCT with involvement of the acetabulum were carefully screened and treated with multiplanar osteotomy and reconstruction using autogenous femoral head implantation combined with total hip replacement. We found that electing the fitting surgical techniques according to the tumor type and boundary is crucial to acquire optimal oncological prognosis. The custom guides for acetabular osteotomies and femoral head reconstruction can produce good functional outcomes with relatively low complications at the intermediate length of follow-up.

Schöllner and Ruck [22] reported the first case of hemi-pelvic prosthesis reconstruction in 1974. Even though the design and materials of the prosthesis are constantly evolving, the long-term survival of these non-biological prostheses remains to be low and associated with a higher rate of prosthesis related complications [7, 17]. Concerning primary pelvic malignant tumors with involvement of the acetabulum, using preoperative and intraoperative imaging and the principle of complete tumor resection, a more precise resection can be achieved. In this way, part of the acetabulum can be retained and autogenous femoral head grafts used for biological reconstruction. Theoretically, applying individually adjusted reconstructive techniques may significantly prolong the survival of the prosthesis, especially for those cases with an anticipated longer survival period. As part of the acetabulum is being retained, more satisfactory postoperative functional outcomes have been reported [23, 24].

In our study, we found that individually adapted resection and reconstruction is superior to hemi-pelvic prosthesis reconstruction, as measured by the MSTS-93 functional score. Bone healing of the pelvic ring significantly improved the long-term survival of the prosthesis compared to a previous study [9]. However, the adaptability of this technique is relatively low and the preoperative and intraoperative imaging as guidance is of particular importance, especially for determining the location of the acetabular osteotomy. Thus, we introduced the use of an intraoperative acetabular osteotomy template according to the preoperative CT and MRI images, which resulted in more accurate and safer resections.

In certain cases of partial oncological acetabular defects, we were able to retain part of the acetabulum according to the preoperative and intraoperative images. Conducting autogenous femoral head transplantation, we reconstructed the acetabular structure to achieve biological comparability.

After resection of a Type A tumor, application of a pelvic reconstructive plate on the remaining upper acetabular margin and pubic ramus was performed to obtain pelvic integrity. After removal of the cartilage of the femoral head and trimming it into suitable bulk, cancellous screws were utilized to fixate the graft onto the acetabulum. The remaining screw holes in the pelvic reconstructive plate were used to fix the graft further to enhance stability. Attention had to be paid to the orientation of the screws, to avoid reaming of the acetabulum. Finally, after reconstructing the new acetabulum, the biological acetabular prosthesis was implanted.

With a Type B tumor, the posterior acetabular defects make it difficult to perform structural bone transplantation. Therefore, we firstly reamed the acetabulum and used the remaining bone to fix and install an acetabular reinforcement ring. We then implanted and fixed the bone graft onto the remaining bone, anterior to the acetabular reinforcement ring. Finally, bone particles were embedded in the gap, followed by insertion of a cemented acetabular cup onto the acetabular reinforcement ring.

The resection technique used for Type C tumors was similar to Type A. In cases with a larger defect of the pelvic area I, the acetabular rotation center needs to be moved slightly upwards to reduce the amount of bone defect. As a result, a distally fixed femoral prosthesis can be selected to increase the offset. This leads to enhancement of the tension of the soft tissues, resulting in increased stability of the joint.

In our study, all bone grafts healed well during the follow-up period, which might be associated with the location of the autogenous cancellous bone and a larger bone contacting area. One case of postoperative displacement of the acetabular prosthesis was associated with initial inadequate compression of the prosthesis, resulting in a failure of bone ingrowth. The initial stability of the hip was also poor in cases with larger soft tissue defects, as two cases experienced early dislocation of the joint. These patients underwent closed reduction under general anesthesia.

Several limitations of our study need to be addressed. Firstly, only a relative proportion of the pubic, ilial or ischial primary malignant tumors with involvement of the acetabulum are candidates for the type of surgery conducted in this study resulting in a small sample size. This may have introduced bias to the results. Secondly, the midterm follow-up period is relatively short for assessment of the oncological prognosis and long-term prosthesis survival. Lastly, we did not compare the performed procedures to normal hemi-pelvic prosthesis reconstruction simultaneously, which makes it difficult to verify whether our technique is superior to alternatives. Despite these limitations, we recruited a total of 20 patients in this retrospective study with an accurate diagnosis, and clinical data and imaging results were fully obtained for analysis. We believe that the performed precise osteotomies might be important for this type of reconstructive strategy to be successful, provided that the type of the primary pelvic malignant tumor is suitable for the chosen procedure. Future studies should be performed using larger samples sizes and a long-term follow-up period, to confirm the advantages of our innovative resection and biological reconstruction techniques.

## Conclusions

Multiplanar osteotomy and biological reconstruction using autogenous femoral head bong-grafting combined with total hip replacement can be performed in certain patients with a primary pelvic malignant tumor involving the acetabulum. According to preoperative and intraoperative imaging, introducing an acetabular template benefits partial acetabular preservation for autogenous graft implantation. The custom guides for acetabular osteotomies and femoral head reconstruction can produce good functional outcomes with relatively low complications and an acceptable rate of local recurrence at the intermediate length of follow-up.

## Data Availability

The dataset supporting the conclusions of this article is available on request– please contact the corresponding author.

## Abbreviations

CT: Computed Tomography
MBGCT: Malignant giant cell tumor of bone
MRI: Magnetic Resonance Imaging
MSTS-93: Musculoskeletal Tumor Society 1993
VAS: Visual analogue scale
